# microRNA regulation of mammalian target of rapamycin expression and activity controls estrogen receptor function and RAD001 sensitivity

**DOI:** 10.1186/1476-4598-13-229

**Published:** 2014-10-06

**Authors:** Elizabeth C Martin, Lyndsay V Rhodes, Steven Elliott, Adrienne E Krebs, Kenneth P Nephew, Erik K Flemington, Bridgette M Collins-Burow, Matthew E Burow

**Affiliations:** Department of Medicine-Section of Hematology and Medical Oncology, Tulane University, New Orleans, LA USA; Department of Pharmacology, Tulane University, New Orleans, LA USA; The Center for Bioenvironmental Research, Tulane University, New Orleans, LA USA; Department of Pathology, Tulane University, New Orleans, LA USA; Medical Sciences and Department of Cellular and Integrative Physiology, Indiana University School of Medicine, Bloomington, IN USA

**Keywords:** miR-155, mTOR, breast cancer, miRNA, Estrogen receptor

## Abstract

**Background:**

The AKT/mammalian target of rapamycin (mTOR) signaling pathway is regulated by 17α-estradiol (E2) signaling and mediates E2-induced proliferation and progesterone receptor (PgR) expression in breast cancer.

**Methods and results:**

Here we use deep sequencing analysis of previously published data from The Cancer Genome Atlas to demonstrate that expression of a key component of mTOR signaling, rapamycin-insensitive companion of mTOR (Rictor), positively correlated with an estrogen receptor-α positive (ERα^+^) breast tumor signature. Through increased microRNA-155 (miR-155) expression in the ERα^+^ breast cancer cells we demonstrate repression of Rictor enhanced activation of mTOR complex 1 (mTORC1) signaling with both qPCR and western blot. miR-155-mediated mTOR signaling resulted in deregulated ERα signaling both in cultured cells *in vitro* and in xenografts *in vivo* in addition to repressed PgR expression and activity. Furthermore we observed that miR-155 enhanced mTORC1 signaling (observed through western blot for increased phosphorylation on mTOR S2448) and induced inhibition of mTORC2 signaling (evident through repressed Rictor and tuberous sclerosis 1 (TSC1) gene expression). mTORC1 induced deregulation of E2 signaling was confirmed using qPCR and the mTORC1-specific inhibitor RAD001. Co-treatment of MCF7 breast cancer cells stably overexpressing miR-155 with RAD001 and E2 restored E2-induced PgR gene expression. RAD001 treatment of SCID/CB17 mice inhibited E2-induced tumorigenesis of the MCF7 miR-155 overexpressing cell line. Finally we demonstrated a strong positive correlation between Rictor and PgR expression and a negative correlation with Raptor expression in Luminal B breast cancer samples, a breast cancer histological subtype known for having an altered ERα-signaling pathway.

**Conclusions:**

miRNA mediated alterations in mTOR and ERα signaling establishes a new mechanism for altered estrogen responses independent of growth factor stimulation.

**Electronic supplementary material:**

The online version of this article (doi:10.1186/1476-4598-13-229) contains supplementary material, which is available to authorized users.

## Introduction

An important downstream mediator of growth factor signaling is mammalian target of rapamycin (mTOR). mTOR is a serine/threonine kinase which belongs to the family of phosphatidylinositol 3-kinase-related kinase protein family [1]. As a regulator of gene translation, mTOR signaling is capable of eliciting a multitude of cellular responses including the regulation of cell growth, proliferation, motility, autophagy, metastasis, and survival. mTOR activation occurs through signaling pathways regulated by insulin like growth factor (IGF), insulin, and nutrient signals [2–5]. The specific effects exerted by mTOR signaling are dependent on the activation of the mTOR complexes mTORC1 and 2, which form intricate negative and positive feedback loops [1, 4]. mTORC1 is a key translational regulator of proteins associated with cell proliferation, metabolism, and growth [1]. The main components of mTORC1 are comprised of regulatory associated protein of TOR (Raptor), proline-rich AKT substrate 40 kDA (PRAS40), Dep domain containing mTOR-interacting protein (Deptor) and lethal with sec13 protein 8 (LST8) [1, 4, 6]. Translation of pro-proliferative and cell growth proteins is induced by mTOR through inhibition of 4E-BP1 and activation of S6K1. mTORC2 is composed of mTOR, mLST8, Sin1, PRR5, Deptor, and Rictor [1, 4, 7]. Interestingly, many of the inhibitors of mTORC1 signaling are activators of mTORC2 signaling. For instance TSC1/TSC2 inhibits mTORC1 signaling through the inhibition of the mTOR activator Ras Homolog Enriched In Brain (Rheb); however, TSC1/TSC2 has been demonstrated to activate mTORC2 signaling through direct contact with mTORC2 [7, 8]. In fact, clinical tumor samples demonstrating a loss of functional TSC1/TSC2 also showed loss of mTORC2 function [9]. In tumor samples, loss of TSC1/TSC2 function resulted in subsequent loss of mTORC2 activity and mTORC1 hyperactivation [10].

mTOR signaling activation by the IGF induced PI3K/AKT pathway has been well described. Crosstalk between mTOR signaling and a number of other important cancer associated signaling pathways, including AMPK/p53, MAPK/ERK, and estrogen receptor-α (ERα) pathways were recently reported [11, 12]. In breast cancer, ERα/mTOR crosstalk is an important indicator of hormone receptor status, as IGF-mediated mTORC1 activation repressed progesterone receptor (PgR) expression in the ER^+^/PgR^−^ breast cancer cell phenotype. This has been characterized by enhanced IGF/mTOR signaling [13, 14]. In addition to regulation of PgR expression, one effector of mTOR signaling, S6 kinase 1 (S6K1), was shown to interact and phosphorylate ERα at serine 167 (S167) following IGF stimulation [15]. Although 17β-estradiol (E2) treatment of breast cancer cell lines induced cell proliferation in an mTOR dependent manner, the molecular mechanism underlying mTOR-mediated ERα signaling in breast cancer remains unclear [16, 17].

Our analysis of TCGA breast tumor data revealed that expression of the mTORC1 activator Rheb strongly correlated with the ERα^−^ phenotype and expression of the mTORC2 signaling component Rictor, correlated with ERα^+^ breast tumor samples. Notably, we observed a positive correlation between Rictor expression and PgR expression levels in the Luminal B molecular subtype. Based on these findings, we investigated the molecular role for mTOR in ERα signaling regulation in breast cancer.

## Results

### mTOR complex 2 signaling correlates with ERα positive breast tumor samples

**Figure 1:**
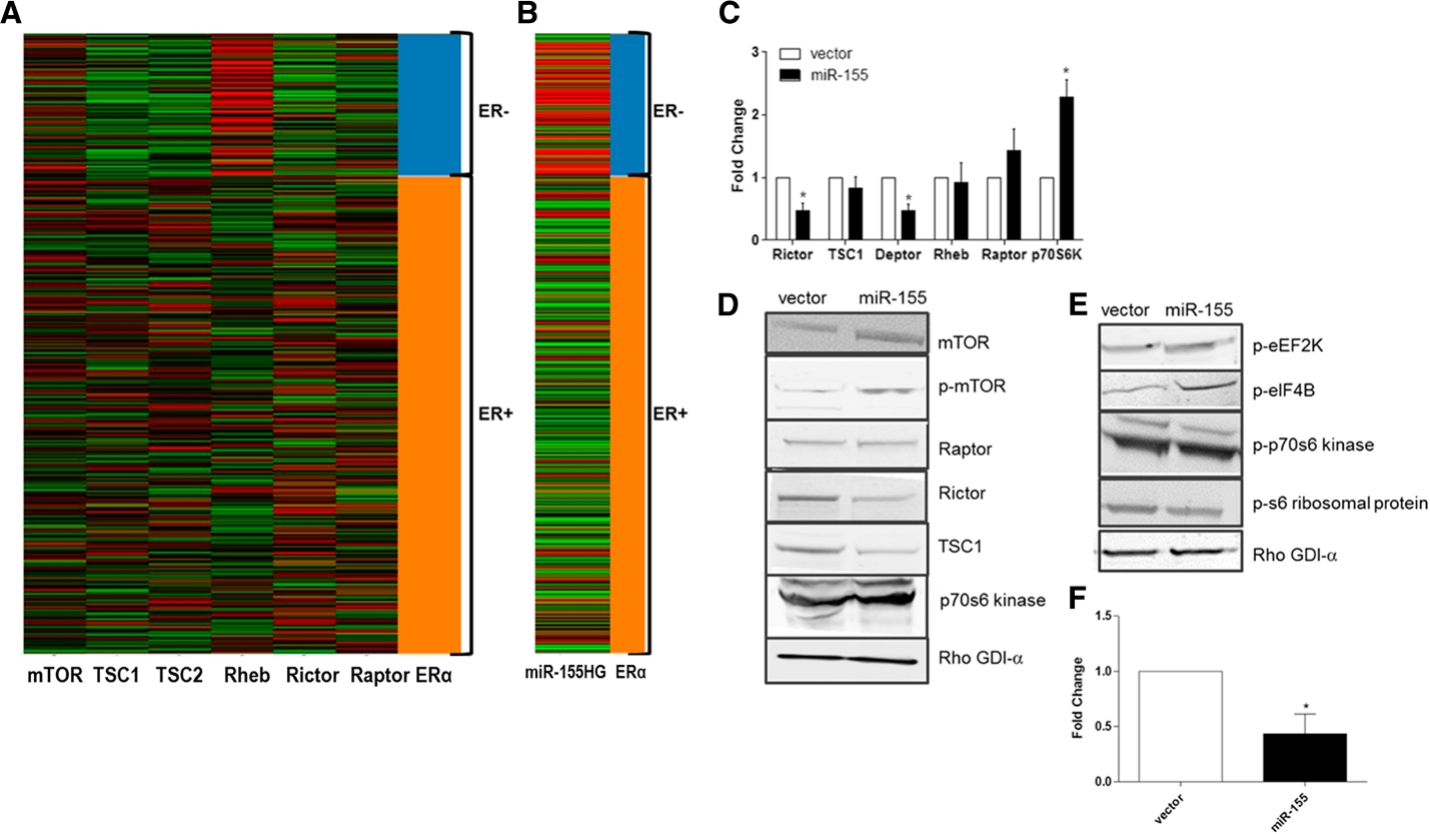
**mTORC1 activation is Associated with an ER Negative Breast Tumor Phenotype.** Deep sequencing data obtained from TCGA data portal was analyzed with respect to ERα (either ERα positive or negative) status for expression of **(A)** mTOR associated gene sets (mTOR, TSC1, TSC2, Rheb, Rictor, Raptor, Rheb) and **(B)** miR-155 host gene (HG) expression. Heat mat depicts high expression (red, +1) and low expression (green, −1). Platform analyzed was TCGA breast invasive carcinoma gene expression IlluminaHiSeq n = 1032. Analysis was sorted based on ERα gene Signature where positive for ER = orange and negative for ER = blue. **(C)** MCF-7-vector and –miR-155 cells were harvested for qPCR analysis for mTOR associated genes Rictor, TSC1, Deptor, Rheb, Raptor, and p70s6 kinase. Ct values were normalized to β-actin and MCF-7-vector cells designated as 1. * p < 0.05 for n ≥ 3. **(D)** MCF-7-vector and –miR-155 cells were harvested for western blot analysis for total and phospho-mTOR (S2448) and mTOR associated proteins Rictor, Raptor, TSC1, and p-70 s6 kinase. Expression normalized to RHO-GDIα, n ≥ 3. **(E)** MCF-7-vector and miR-155 cells were harvested for western blot analysis for phosphorylation of downstream mTORC1 associated proteins p-eIF4B (S422), p-S6 ribosomal protein (S235/236), p-p70s6 kinase (Thr389), p-eEF2K (S366). Expression was normalized to RHO-GDIα, n = 2. **(F)** qPCR of MCF-7-vector and miR-155 cells for downstream mTORC2 regulated gene PKCα, normalization was to β-actin and vector designated as 1, n = 3.

mTOR signaling is emerging as a prominent mediator of cancer progression enhancing both proliferation and metastasis [4, 5]. Due to the divergent roles played by mTOR signaling complexes mTORC1 and mTORC2 we sought to determine the expression levels of key mTOR signaling components in a cohort of breast cancer tumor samples. The Cancer Genome Atlas (TCGA) deep sequencing data of breast cancer invasive carcinoma gene expression (IlluminaHiSeq) was analyzed and viewed in the UCSC Cancer Genomics Browser [18–21]. The ERα gene signature was used to filter expression levels in tumor samples (either ERα-positive or ERα-negative) and the mTOR associated genes Rictor, Raptor, Rheb, TSC1, TSC2, and mTOR were analyzed. Results demonstrate that Rictor, TSC1, and TSC2 (all activators of mTORC2 signaling) have higher expression levels in ERα^+^ tumor samples compared to ER^−^ (Figure 1A). Interestingly, mTORC1 signaling components had varied expression with respect to ERα expression. Rheb had high expression levels correlating to ER^−^ tumors while Raptor demonstrated higher expression levels in ERα^+^ tumors (Figure 1A). There was no observed correlation for mTOR expression with either an ERα^+^ or ERα^−^ breast cancer phenotype. As both Raptor and Rictor show a positive correlation with ERα expression we then used the Breast Cancer Gene Expression Miner v3.0 and further examined the correlation between Raptor or Rictor expression with ERα expression [22]. Positive correlations for both Rictor and Raptor with ERα expression were observed, however there was a stronger correlation between Rictor and ERα (Pearson’s correlation coefficient r = 0.32) than Raptor and the ERα (Pearson’s correlation coefficient r = 0.20) (Additional file 1: Figure S1A and Additional file 1: S1B). As TSC1/TSC2 complex is an activator of mTORC2 signaling and a repressor of mTORC1 and Rheb is an activator of mTORC1 signaling, this data suggests that mTORC2 signaling may be more prominent in ERα^+^ and mTORC1 signaling may be more prevalent in ERα^−^ breast carcinomas.

We previously demonstrated that miRNA expression was a target of IGF/AKT signaling [23] suggesting a potential for miRNA crosstalk in the regulation of mTOR signaling. This led us to next test if miRNAs could represent regulators of the differential expression profiles of the mTORC signaling components. To examine the molecular mechanism underlying loss of Rictor and Raptor expression observed in ERα^−^ breast tumors, putative miRNA target sites in the 3’UTR of both genes were analyzed using TargetScan6.0. Analysis of highly conserved miRNAs demonstrated that while several binding sites for multiple miRNAs were apparent in the 3’UTR of Rictor, Raptor appeared to be targeted by only five highly conserved miRNAs (Additional file 2: Tables S1 and Additional file 3: Table S2). Due to the potential for more miRNAs to target Rictor, we therefore investigated miRNAs predicted to target Rictor in an ERα^+^ breast cancer cell line. The miRNAs (miR-203, miR-194, miR-98, let-7 g, and miR-155) predicted to have seed sites in the 3’UTR of Rictor were stably over expressed in the ERα^+^ MCF-7 cell line and screened by qPCR for Rictor expression levels. Of these five miRNAs, only miR-155, a miRNA well known for playing various roles in cancer, was capable of significantly inhibiting Rictor expression (Additional file 4: Figure S2A).

### miR-155 enhances mTOR activity by targeting members of AKT/mTOR signaling pathway

To better evaluate a role for miR-155 expression with respect to mTOR signaling and the ERα gene signature, we next analyzed expression of the miR-155 host gene (miR-155HG) across TCGA tumor data. In opposition to Rictor expression, the miR-155HG, which encodes the mature miR-155 sequence, correlated with an ERα^−^ status in TCGA breast tumor samples and mature miR-155 expression correlated with an ERα^−^ status in breast cancer cell lines (Figure 1B and Additional file 4: Figure S2B). As miR-155 expression correlated an ERα^−^ phenotype and Rictor expression correlated with ERα^+^ tumors, we next investigated whether the observed high levels of miR-155 expression in ERα^−^ breast cancers was a driving force for the repression of Rictor. The MDA-MB-157 breast cancer cell line demonstrated the highest levels of miR-155 expression (Additional file 4: Figure S2B), so we chose this cell line and transfected a doxorubicin inducible red fluorescent protein (RFP)-miR-155sponge designed to inhibit miR-155 expression. Following transfection and RFP induction, we performed qPCR to determine Rictor expression levels. qPCR was performed and results demonstrated an increase (p < 0.06) in Rictor expression levels following miR-155 inhibition (Additional file 4: Figure S2C). In order to investigate the relationship between miR-155, mTOR, and ERα signaling; we used the ER^+^ MCF-7 cell line transfected with miR-155 as this cell line demonstrated repressed Rictor expression levels and expressed levels of miR-155 equivalent to that of ER^−^ cell lines (Additional file 4: Figure S2A and S2D respectively). To better understand the relationship between miR-155 expression and the mTOR signaling cascade, we uploaded all miR-155 predicted targets using Pathway Interaction Database (PID) [24] and obtained network maps for predicted miR-155 target genes and pathways (Table 1). Strikingly, many of these pathways were mediated by PI3K signaling or growth factors, which have been shown to crosstalk with mTOR signaling and indeed many components of both mTOR signaling complexes (mTORC1 and 2) were, predicted targets of miR-155 (Table 1) [25].

**Table 1:** **Pathways Predicted to be altered by miR**-**155 Target Regulation**

Pathway	p value
PDGFR-beta signaling pathway	2.77E-11
TGF-beta receptor signaling	1.74E-09
Signaling events mediated by hepatocyte growth factor receptor (c-Met)	6.00E-09
IL4-mediated signaling events	9.61E-08
CXCR4-mediated signaling events	2.22E-08
mTOR signaling pathway	4.37E-08
IGF1 pathway	7.71E-08
Regulation of retinoblastoma protein	9.30E-08
Signaling events mediated by stem cell factor receptor (c-Kit)	2.75E-07
AP-1 transcription factor network	2.76E-07
ErbB1 downstream signaling	3.70E-07
Neurotrophic factor-mediated Trk receptor signaling	5.56E-07
Direct p53 effectors	5.80E-07
FGF signaling pathway	6.75E-07
GMCSF-mediated signaling events	7.06E-07

p value determined by size of miR-155 target data set, number of molecules in a pathway, and number of molecules in database. Determines probability that miR-155 targets are biased towards a particular pathway.

Given that the TCGA tumor data demonstrated an inverse relationship between the loss of Rictor expression and miR-155HG expression in relation to ERα status and that Rictor expression was repressed in our MCF7-miR155 cell line, we next sought to determine the effects of miR-155 on mTOR signaling. By combining our in-house Seedfinder program (identifies isoform specific seedsites across the genome for miR-155) with previously published deep sequencing data for MCF-7 cells and the UCSC Genome Browser [26, 27]. Appropriate miR-155 targets were chosen for further investigation based on evaluation of isoforms with 3’UTR being expressed in MCF-7 cell line (Table 2). We determined that the p70s6K 3’UTR possessed an 8-mer site its 3’UTR and the 3’UTRs of Deptor, Rheb, and TSC1 each possessed 7-mer sites (Table 2). Based on this, qPCR was performed for Deptor, Rheb, TSC1, Raptor, and p70s6K. Results demonstrate that in MCF-7-miR-155 cells, significantly increased p70s6 kinase expression was observed (Figure 1C), and significantly decreased expression of the mTOR repressor Deptor was seen (Figure 1C). Western blot analysis further confirmed increased mTOR activity demonstrated through the increased total and phospho-mTOR (S2448) in MCF-7-miR-155 cells (Figure 1D). In addition, decreased Rictor and TSC1 protein levels were observed in MCF-7-miR-155 cells (Figure 1D). The combined loss of Rictor (a critical mTORC2 component) and TSC1an mTORC2 activator and mTORC1 suppressor) suggests that miR-155 induced mTOR signaling through the mTORC1 complex. Evaluation of downstream targets of mTORC1 and mTORC2 were next evaluated. Western blot demonstrated enhanced phosphorylation of p-eEF2K and p-eIF4B downstream targets of mTORC1 (Figure 1E), there was however no noticeable change in p-p70s6K or p-S6 ribosomal protein. mTORC2 is known to enhance PKCα expression, so we next evaluated PKCα gene expression and saw repressed expression of PKCα in the MCF-7-miR-155 cell line (Figure 1F).

**Table 2:** **mTOR Associated miR**-**155 Target Sequences Expressed in MCF**-**7 Brest Cancer Cells**

Gene	Isoforms	MCF-7 isoforms with miR-155 target sites	8-mer sites	7-mer1A sites	7-mer8 sites
Rheb	1	1	0	1	0
TSC1	8	3	0	1	0
p70s6K	6	5	1	0	1
Rictor	5	3	1	0	1
PRKAA2	1	1	1	0	1
PML	17	3	0	0	1
EEF2	1	1	0	1	0
Deptor	2	2	0	1	0
EIF4E	4	3	0	1	0
ULK2	2	1	0	1	0
YWHAE	4	4	0	0	1

### Stable expression of miR-155 disrupts ERα signaling in MCF-7 breast cancer cells

**Figure 2:**
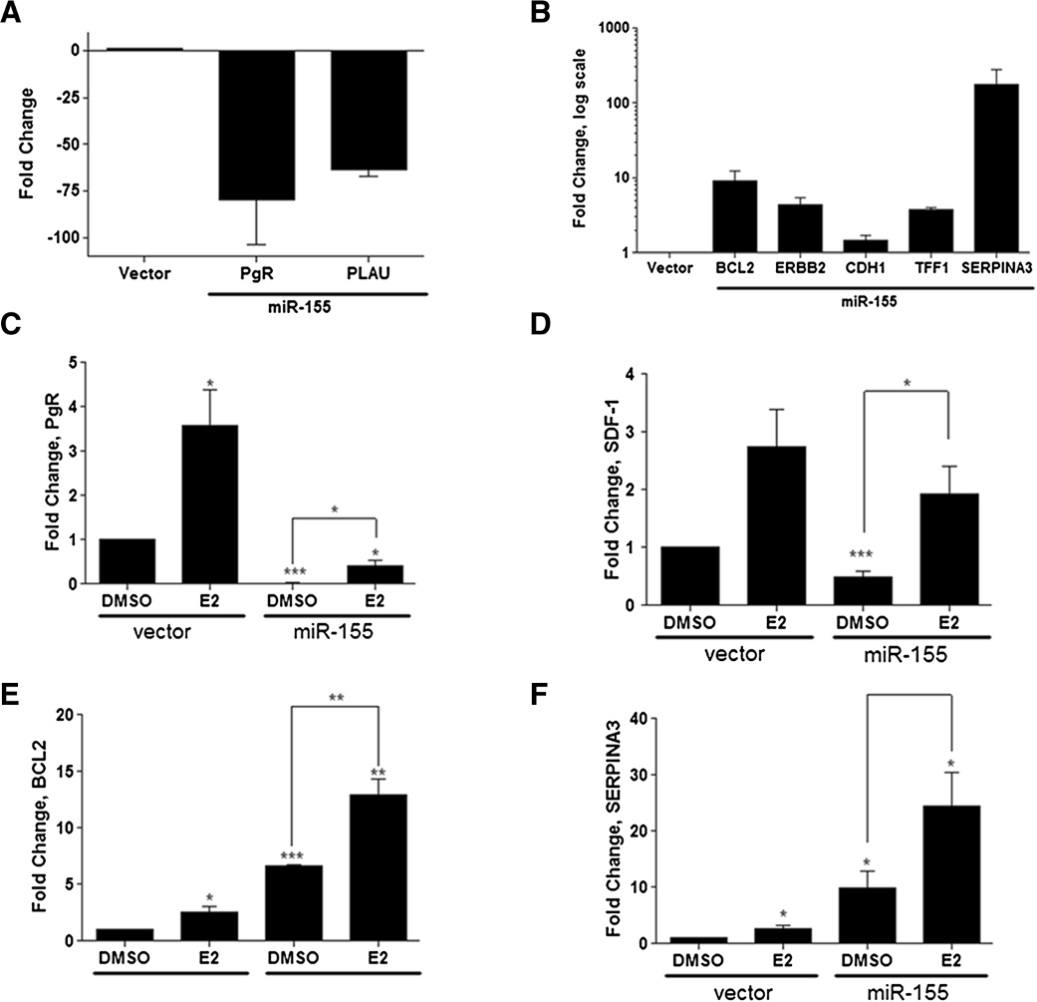
**Overexpression of miR-155 selectively alters estrogen stimulated gene expression of ER-regulated genes** ***in vitro*** **. (A-B)** Gene panel array for ER regulated and cancer associated genes, n = 2. **(A)** Down regulated genes and **(B)** up regulated genes (logarithmic scale) in MCF-7-miR-155 cells compared to vector control. Results represent average fold change ± SEM. **(C-F)** QPCR was performed for genes, n = 5 **(C)** PgR, **(D)** SDF-1, **(E)** BCL2, and **(F)** SERPINA3. Cells were grown in 5% CS phenol free DMEM for 48 hours before treatment with DMSO or E2 (1nM) for 24 hours. Cycle number was normalized to beta actin and MCF-7-vector control cells treated with DMSO scaled to 1. Bars represent average fold change ± SEM. * p < 0.05; ** p < 0.01; *** p < 0.001.

As crosstalk between mTOR signaling and ERα has been reported [13, 14], it was of interest to investigate a possible role for miR-155 in ERα signaling. We performed qPCR with an ERα-responsive and breast cancer associated genes qPCR gene array. Aberrant basal expression of ERα-regulated genes was observed in the MCF7-miR-155 cell line compared to vector. Altered genes included PgR (a known target of ERα and mTOR signaling) and PLAU (both decreased) and BCL2, ERBB2, TFF1, and SERPINA3 (all increased) (Figure 2A and Figure 2B respectively). This striking divergent expression of ERα-regulated genes suggested that miR-155 acts as a possible regulator of estrogen-mediated signaling. To further investigate this possibility, cells were serum starved for 48 hours, treated with 17-α estradiol (E2, 1 nM) or vehicle for 24 hours, and ERα target genes (PgR, SDF-1, BCL2, and SERPINA3) were analyzed by qPCR. As previously observed in our gene panel array, significantly decreased basal PgR mRNA levels were observed in MCF-7-miR-155 cells compared to MCF-7-vector (Figure 2C). Basal expression of SDF-1 was also significantly lower in our MCF-7-miR-155 cell line (Figure 2D). Basal expression levels of BCL2 and SERPINA3 were significantly increased in MCF-7-miR-155 cells and E2 treatment further induced expression of both genes compared to MCF-7-vector cells (Figure 2E and Figure 2F respectively). E2 stimulation failed to increase PgR expression levels in MCF-7-miR-155 cell line to that of basal levels observed in the MCF-7-vector cell line. It should be noted that PgR, along with SDF-1, BCL2, and SERPINA3 were all increased following E2 stimulation; however, PgR alone remained significantly repressed following E2 stimulation. Based on these results, we conclude that overexpression of miR-155 in ERα^+^ breast cancer cells disrupted E2 signaling but did not completely inhibit the cellular response to hormone.

### miR-155 induced mTOR/ERα crosstalk is not through direct mTOR induced phosphorylation of ERα

Since PgR was the only E2 responsive gene that remained significantly repressed and mTOR is a known mediator of ER signaling both directly and indirectly, we next set out to further define the effects of miR-155 expression on mTOR/ERα crosstalk by evaluating ERα expression levels and PgR protein levels and function. Following qPCR, there was no difference in basal ERα mRNA or protein levels observed between the MCF-7-miR-155 cells versus control (Additional file 5: Figure S3A and S3B respectively). As mTOR signaling is known to activate ERα phosphorylation at S167 we next sought to evaluate ERα phospho-levels for S167. Western blot demonstrates a loss of ERα phosphorylation at S167 (Additional file 5: Figure S3B), suggesting mTOR activation is not increasing ERα activity directly. Western blot revealed basal PgR protein levels were decreased in MCF-7-miR-155 cells compared to -vector cells (Additional file 5: Figure S3B). To assess PgR functional activity, a progesterone response element (PRE)-luciferase assay was performed. MCF-7-vector and -miR-155 cells were transfected with a PRE-luciferase construct and treated with progesterone in a dose dependent manner. The doses of progesterone (100 nM, 1 μM, 10 μM but not 10 nM) significantly increased PRE activity in MCF-7-vector cells. MCF-7-miR-155 cells demonstrated lower levels of PRE activity both basally and after 10 nM progesterone treatment compared to MCF-7-vector cells (Additional file 5: Figure S3D). PRE-activity in MCF-7-miR-155 cells was similar to that of basal unstimulated levels of MCF-7-vector cells for the 100 nM, 1 μM, 10 μM doses of progesterone. Stimulation of PgR with 10 nM E2 for 24 hours prior to treatment with progesterone was similar to progesterone alone, with MCF-7-miR-155 cells demonstrating a loss of PgR activity (Additional file 5: Figure S3E). Given the loss of functional PgR in MCF7-miR155 cells compared to -vector and no observed increase in phospho-ERα (S167) in the MCF-7-miR-155 cell line, we suggest that miR-155-induced ERα signaling regulation was due to the loss of Rictor expression rather than direct ERα-mTORC1 cascade interactions with ERα.

### miR-155 augments E2-stimulated proliferation *in vitro*and *in vivo*

**Figure 3:**
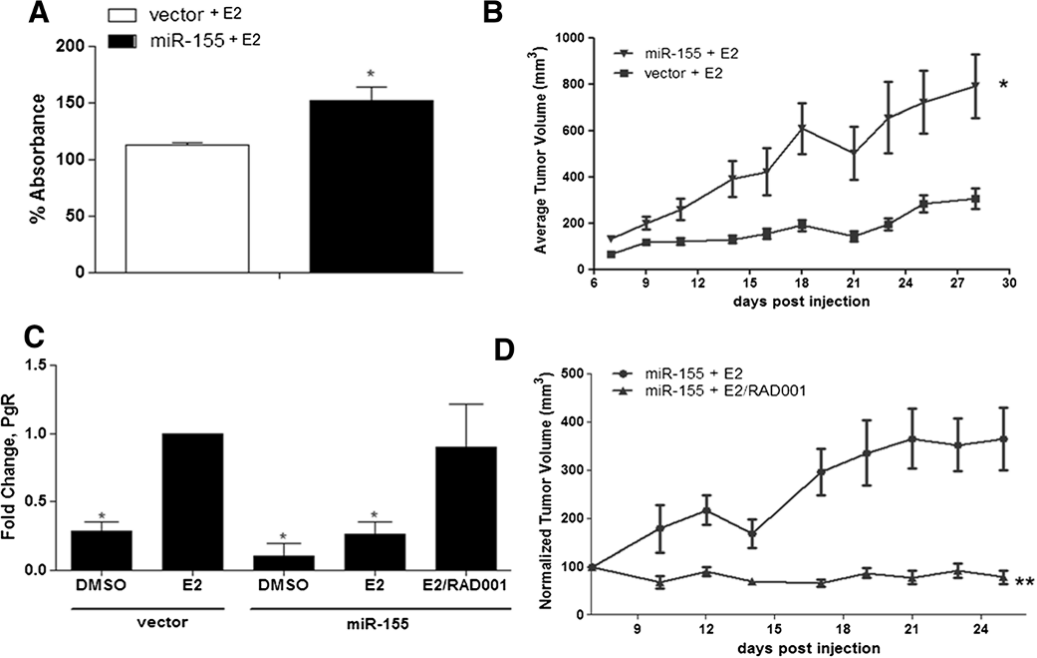
**miR-155 enhanced E2 stimulated proliferation is mediated through altered mTOR signaling** ***in vivo*** **and** ***in vitro*** **. (A)** Crystal violet assay for proliferation of MCF-7-vector and MCF-7-miR-155 cells treated with E2. Cells were grown in 5% CS phenol free DMEM for 48 hours prior to treatment with E2 (1nM) or vehicle control (DMSO). Each cell line was normalized to the respective vehicle control, n = 4. **(B)** Tumor volume for ovariectomized CB-17 SCID female mice injected bilaterally with 5X10^^6^ MCF-7-vector cells or MCF-7-miR-155 cells, n = 5 animals/group. All animals were implanted with an E2 pellet (0.72 mg) at day of cell injection. Points represent average tumor volume ± SEM. **(C)** MCF-7-vector and –miR-155 cells were harvested for total RNA extraction and PCR was performed for PgR following treatment with E2 (1nM), RAD001 (20nM) + E2, or vehicle control (DMSO), normalized to MCF-7-vector treated with E2 (1nM). Bars represent fold change ± SEM, n ≥ 3. * Significantly different from MCF-7-vector + E2 p < 0.05. **(D)** Tumor volume for ovariectomized CB-17 SCID female mice injected bilaterally with 5X10^^6^ MCF-7-miR-155 cells + E2 + RAD001 vs. MCF-7-miR-155 + E2 given placebo, n = 7. Points represent normalized tumor volume ± SEM. * p < 0.05, ** p < 0.01.

Because miR-155 altered basal ERα-mediated gene expression (Figure 2A and Figure 2B) and maintained suppression of the E2 responsive gene PgR (Figure 2C), we sought to determine the biological consequence of miR-155-altered E2 stimulation. MCF-7-miR-155 and –vector cells were serum starved for 48 hours prior to stimulation with 1 nM E2 for 72 hours. Cell proliferation was assessed using crystal violet assays. Treatment with E2 stimulated proliferation of both the MCF-7-vector and MCF-7-miR-155 cell lines (Figure 3A); however, E2-stimulated proliferation was significantly greater in MCF-7-miR-155 cells versus MCF-7-vector cells (52 ± 11.94% versus 12.8 ± 2.62%). To determine if the enhanced E2 response increased tumorigenesis *in vivo*, ovariectomized CB-17/SCID female mice were inoculated with either MCF-7-vector or -miR-155 cells in the mammary fat pad (MFP) in the presence of exogenous E2 (0.72 mg pellet, 60 day release) versus placebo. At necropsy (day 28 post cell injection), final tumor volume was significantly greater for MCF-7-mir-155 tumors (791.96 ± 137.45 mm3) compared to vector tumors (306.12 ± 44.85 mm3) (Figure 3B). These results together demonstrated *in vivo* and *in vitro* that miR-155 expression enhances estrogen response.

### miR-155 inhibition of PgR expression is regulated through mTORC1 activation

Given that miR-155 induced enhanced E2 stimulated tumorigenesis and proliferation while simultaneously repressing PgR we next set out to investigate whether miR-155 activation of mTORC1 leads to the suppression of PgR. qPCR analysis was conducted after treatment of MCF-7-miR-155 with 1 nM E2 and the mTORC1 specific inhibitor RAD001 (20 nM). qPCR results revealed a significant increase in PgR expression in MCF-7-miR-155 cells following the combined RAD001/E2 treatment and PgR levels were equal to that of MCF-7-vector cells treated with E2 only (Figure 3C). Additionally, induction of PgR in MCF-7-miR-155 cells by RAD001 and E2 was significantly greater than E2 only treatment (Figure 3C). PgR expression in MCF-7-vector cells treated with both RAD001 and E2 was not significantly different compared to E2 treatment alone (data not shown). Collectively, the data indicated that miR-155 induced regulation of mTORC1 activity in MCF-7-miR-155 cells inhibited PgR expression. To validate the activity of RAD001, we performed western blot analysis for the eukaryotic translation initiation factor 4E binding protein (4E-BP1) and Akt (S473) phosphorylation levels, downstream effector and target proteins of the mTORC1 complex. MCF-7-vector and MCF-7-miR-155 cells were treated with the mTORC1 specific inhibitor RAD001. As anticipated, phosphorylation of 4E-BP1 was decreased and Akt, which is inhibited by mTORC1 activity, was increased in MCF-7-miR-155 cells following treatment with RAD001 (Additional file 6: Figure S4).

As mTOR signaling is known to require E2 induced proliferation we next sought to determine if mTOR signaling was involved in the heightened E2 induced tumorigenesis observed in our MCF-7-miR-155 cells. To test this CB-17/SCID ovariectomized mice were inoculated with MCF-7-miR-155 cells in the presence of E2 (0.72 mg pellet, 60 day release). Mice were administered 5 mg/kg/day of RAD001 or vehicle daily following palpable tumor formation (day 7 post cell injection), and tumor size was recorded every two to three days until necropsy on day 25. The inhibitory effect of RAD001 was apparent by day 10 post injections, MCF-7-miR-155 tumors in vehicle-treated animals increased to 364.15% ± 65.07% mm3 at Day 25 from Day 7 (100%). In contrast, treatment with RAD001 rapidly decreased tumor size (78.23% ± 13.77% mm3; Figure 3D), and significant inhibition of E2-stimulated tumorigenesis continued through day 25 post injection (study terminated due to the large tumor size of the vehicle control group per approved animal protocol). Taken together these results further support a role for miR-155 induced mTOR-ERα crosstalk *in vitro* and *in vivo*.

### Luminal B molecular subtypes divergent expression of mTOR signaling components and PgR expression

**Figure 4:**
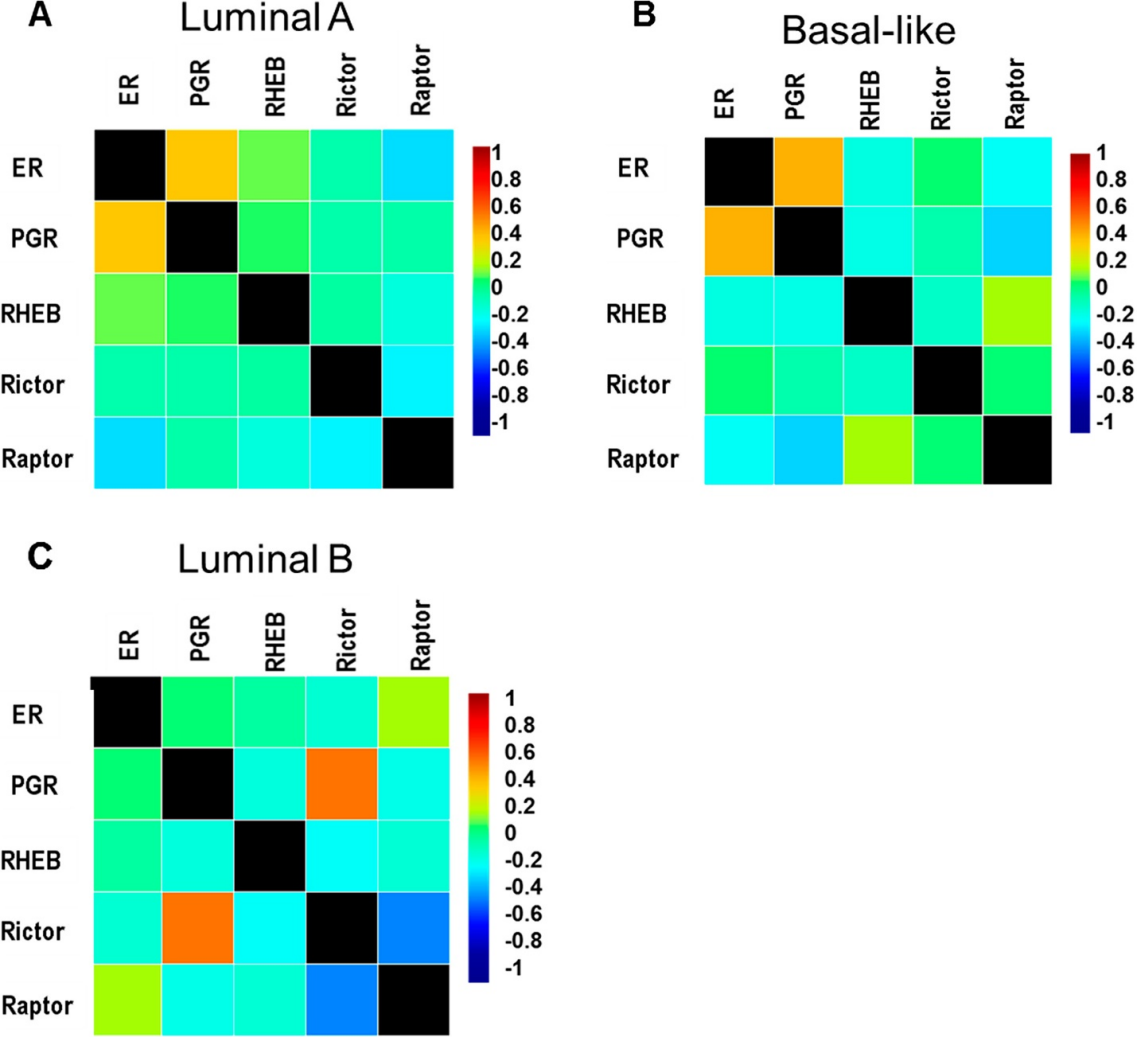
**Rictor expression positively correlates with PgR expression in Luminal B breast cancer molecular subtype. (A-C)** Targeted gene expression correlation analysis for ER, PgR, Rictor, Rheb, and Raptor derived from pooled breast cancer samples obtained from BC-GenExMiner-v3.0. Correlation maps generated based on breast cancer molecular subtype. **(A)** luminal A, **(B)** Basal-like, **(C)** luminal B.

As increased activation of mTORC1 is known to mediate PgR expression and we demonstrated that loss of Rictor expression correlated with an ERα^−^ breast cancer phenotype, we next sought to determine if there was a clinical correlation between Rictor or Raptor expression and loss of PgR expression. Genomic data obtained through the Breast Cancer Gene Expression Miner v3.0 was analyzed for mTOR signaling components (Rictor, Raptor, and Rheb), ERα, and PgR[22]. Rheb expression was included in this analysis as it is an activator of mTORC1 signaling and demonstrated high expression levels in the TCGA ERα breast cancer tumors (Figure 1A). No correlation between Rictor and PgR expression was observed in either the ERα^+^ luminal A or basal-like tumor profiles (Figure 4A and Figure 4B). However, since MCF-7-miR-155 cell line maintained an ERα^+^ phenotype with altered ERα signaling (evident through loss of PgR and high levels of TFF1, Figure 2A and Figure 2B), we next sough to determine the correlation between Rictor and PgR in a luminal B tumor subtype. As seen in Figure 4C, Rictor expression significantly correlated with PgR expression (Pearson’s correlation coefficient r = 54) and was inversely related to Raptor expression (Pearson’s correlation coefficient r = −0.41) in breast cancers possessing a luminal B subtype.

## Discussion

The luminal B breast cancer subtype is classified as ERα^+^; however, altered ER signaling is commonly observed along with loss of PgR expression. Additionally this subtype represents a more aggressive stage of disease than the luminal A subtype and has the potential to progress to endocrine resistance and hormone independence [13, 28, 29]. Here we demonstrate a clinical correlation between the mTORC2 signaling component Rictor and receptor status where Rictor expression correlates positively with expression of both ERα and PgR expression. Additionally like others, we demonstrate a link too receptor status and miR-155 expression [30]. Through miR-155 overexpression in the ER^+^ MCF-7 breast cancer cell line we demonstrate alterations in the mTOR signaling cascade can result in the loss of PgR expression without prior growth factor stimulation. Previous studies have shown loss of PgR expression in clinical samples is used as an indicator of aberrant growth factor signaling and the IGF induced AKT/mTOR signaling pathway is commonly associated with the repression of PgR in breast cancer systems. Additionally inhibition of mTOR signaling has been shown by others to result in a loss of ERα-mediated gene transcription [15]. While these studies have demonstrated activation of mTORC1 signaling by IGF as a regulatory mechanism for PgR repression, our results suggest that both miR-155 and Rictor may be important mediators of mTORC1 activity and PgR expression irrespective of growth factor stimulation. In support, others have shown loss of Rictor enhanced signaling of mTORC1 while increased expression of Rictor led to in inhibition of mTORC1-mediated signaling. It was suggested that these results are due to a change in the availability of the mTOR protein for mTORC complex assembly [31, 32]. Our data suggests that loss of Rictor may induce mTORC1 activity and thus PgR suppression, as we see mTORC1 signaling-dependent inhibition of PgR expression. This is evident through our *in vitro* and *in vivo* experiments using the mTORC1 specific inhibitor to induce PgR expression following treatment with E2 and to inhibit E2-stimulated tumorigenesis. Current studies show a link between mTOR and E2-induced tumorigenesis and cellular proliferation where RAD001 is capable of suppressing E2-induced tumor growth and cellular proliferation [15, 33]. Additionally a synergistic relationship exists between treatment of ER^+^ breast cancers with endocrine therapies and mTOR inhibitors in breast cancer cell lines.

**Figure 5:**
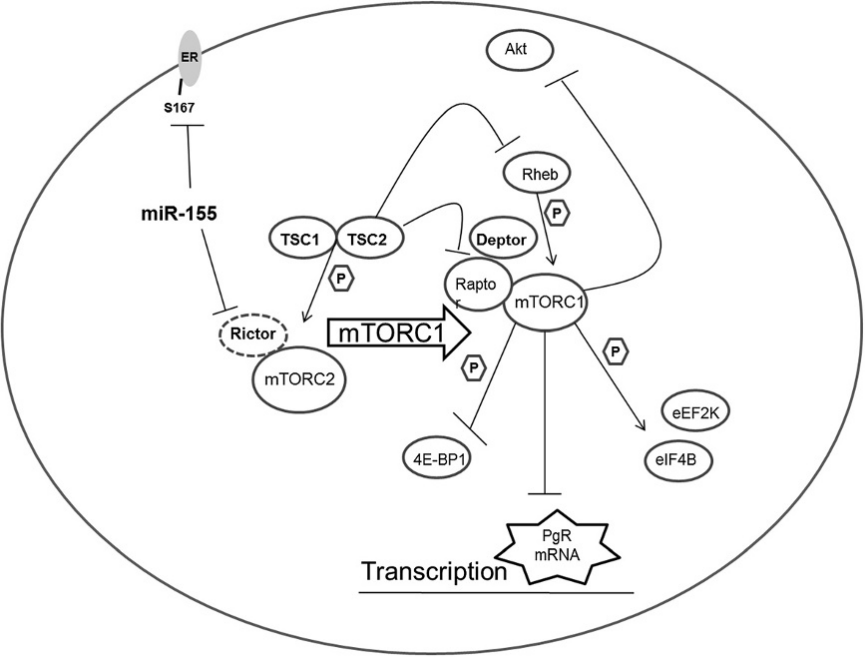
**mTORC1 induced repression of PgR is regulated by miR-155 independently of growth factor stimulation.** Schematic for miR-155 induced regulation of mTOR/ER crosstalk.

Taken together, our data demonstrate a role for a miR-155-mTOR-ERα signaling axis in the progression of breast carcinomas towards a hormone independent phenotype evident through the loss of PgR (Figure 5). Numerous studies have recently shown that miRNAs can act as mediators of ERα signaling, either by direct targeting of ERα for degradation or through inhibition of molecules pertinent to the ERα pathway [34–36]. Additionally it has recently been demonstrated by Zhang *et al*. that E2 is a positive regulator of miR-155 expression in the MCF-7 breast cancer cell line [37]. miR-155 is a frequently deregulated miRNA in human breast cancers and increases cellular proliferation in breast cancer cell lines [38]. Our data and others demonstrate increased miR-155 expression correlates with an ERα^−^ status in human breast tumor subtypes as well as breast cancer cell lines [30, 39, 40]. We extend previous studies by showing that miR-155 expression alters hormone receptor signaling and expression of the ERα regulated gene, PgR, through alterations in the mTOR signaling pathway. While previous studies have demonstrated miR-155 to be an inhibitor of mTORC1 signaling through the suppression of Rheb in macrophages [41], we do not see loss of Rheb expression in our breast cancer cell line and instead see an inhibition of mTORC2 signaling components. miR-155 has recently be shown to target multiple aspects of the mTOR signaling cascade, including mTORC2 component Rictor; however, our results are the first to demonstrate miR-155 induced mTOR/ER crosstalk through enhanced mTOR signaling and Rictor suppression [42]. Additionally our study shows miR-155 expression induces increased phosphorylation of downstream mTORC1 proteins associated with translation but not through the classically activated mTOR/p70s6k pathway. Taken together this suggests miR-155 inhibition and activation of mTOR components to be cell line specific. Finally our analysis of clinical data shows a strong correlation between mTOR and ERα signaling cross talk in luminal B breast cancer, as this subtype showed a positive correlation for Rictor and PgR expression, supporting the need for further molecular studies on the inverse relationship between Raptor and Rictor in breast cancer.

## Materials and methods

### Cells and reagents

MCF-7, MDA-MB-157, and BT-549 human breast cancer cell lines were acquired from American Type Culture Collection (Manassas, VA). Liquid nitrogen stocks were made upon receipt and maintained until the start of study. ERE–luciferase and/or qPCR for ER and PgR were used to confirm MCF-7 sustained estrogen responsiveness. Morphology and doubling times were also recorded regularly to ensure maintenance of phenotype for all cell lines. Cells were used for no more than 6 months after being thawed. Cells were maintained as previously described [43]. RAD001 was purchased from Selleck Chemicals LLC, and 17-beta Estradiol (E2) from Sigma (Sigma-Aldrich St. Louis, MO).

### Animals

4–6 wks. old ovariectomized SCID/CB17 female mice (Charles River Laboratories; Wilmington, MA) were allowed a period of adaptation in a sterile and pathogen-free environment with food and water *ad libitum*. Cells were harvested in the exponential growth phase using a PBS/EDTA solution and washed. Viable cells (5 × 10^6^) in 50 μl of sterile PBS suspension were mixed with 100 μl Reduced Growth Factor Matrigel (BD Biosciences, Bedford, MA). Injections were administered into the mammary fat pad using 27 ½ gauge sterile syringes. Animals were divided into treatment groups of five mice each: MCF-7 control vector, MCF-7 control vector plus E2, MCF-7 cells transduced to overexpress mature miR-155, MCF-7 cells transduced to overexpress mature miR-155 plus E2. Placebo or E2 pellets (0.72 mg of estradiol-17α, 60-day release; Innovative Research of America; Sarasota, FL) were implanted subcutaneously in the lateral area of the neck using a precision trochar (10 gauge). All procedures in animals were carried out under anesthesia using a mix of isofluorane and oxygen. RAD001 (Everolimus) (Selleck Chemicals LLC, Houston TX) was administered as a micro emulsion dissolved in sugar water as 5 mg/kg/day. Tumor size was measured every 2–3 days using digital calipers. The volume of the tumor was calculated using the formula: 4/3π LS2 (L = larger radius; S = shorter radius). Animals were euthanized by cervical dislocation after exposure to CO_2_. Tumors were removed and frozen in liquid nitrogen or fixed in 10% formalin for further analysis. All procedures involving these animals were conducted in compliance with State and Federal laws, standards of the U.S. Department of Health and Human Services, and guidelines established by Tulane University Animal Care and Use Committee. The facilities and laboratory animals program of Tulane University are accredited by the Association for the Assessment and Accreditation of Laboratory Animal Care.

### RNA Extraction and Quantitative Real Time RT-PCR

MCF-7-vector and MCF-7-miR-155 cells were harvested for total RNA extraction using Qiagen RNeasy RNA purification system or for microRNA miRNeasy purification system per manufacturer’s protocol (Qiagen, Valencia, CA). Quantity and quality of the RNA and miRNA were determined by absorbance at 260 and 280 nm using the NanoDrop ND-1000. 2 ug of total RNA was reverse-transcribed using the iScript kit (BioRad Laboratories, Hercules, CA) and qPCR was performed using SYBR-green (Bio-Rad Laboratories, Hercules, CA). β-Actin, PgR, ERα, BCL-2, SDF-1, SERPIN3A, Rictor, TSC1, Raptor, Deptor, p70s6 kinase, and Rheb genes were amplified n > 3. E2 stimulation experiments, cells were grown in 5% DMEM for 48 hours prior to treatment with 1 nM E2 or DMSO for 24 hours. RAD001 *in vitro* experiments cells were pre-treated for 30 minutes with 20 nM RAD001 followed by 100 pM E2 or DMSO. miRNA was reverse–transcribed using the SABiosciences RT^2^ miRNA first strand kit (Qiagen, Valencia, CA) and qPCR was performed using SABiosciences SYBR green, miR-155 primer, U6 primer, and SA- Bioscience RT^2^ cancer miRNA array plate (MAH-102A) were purchased from Qiagen (Valencia, CA). Data was analyzed by comparing relative target gene expression to β-actin for mRNA and U6 for miRNA. Relative gene expression was analyzed using 2-ΔΔCt method [44].

### Transfection of Cell Lines

miR-155 and vector plasmid were generated as previously described[45]. MCF-7 cells were transfected with pre-mir-155 or vector plasmid using Lipofectamine 2000 at 1ug/ul OPTI-MEM (Invitrogen, Grand Isles, NY) as per manufacturer’s protocol. Parental MCF-7 cells were grown in a 100 mm dish. 5ug pre-mir-155 or vector plasmid was added to 100 ul serum free opti-MEM then 15 ul Lipofectamine was added. Following 30 minutes opti-MEM containing plasmid was added to MCF-7 cells. The following day cells were treated with 300 ng/ml puromycin. Cells were maintained in 10% DMEM and treated with 300 ng/ml puromycin every two days for 2 weeks. Colonies were pooled and verification of mature miR-155 overexpression was confirmed using qPCR for mature miR-155. Stable pools were maintained in 10% DMEM as described above. For generation of miR-155 sponge, miR-155 sponge sequence was taken from pMSCV-puro-GFP-miR155SPONGE as previously described [46] and inserted downstream from the RFP sequence in the TRIPz-RFP vector backbone. Sponge was transfected through lenti-viral transfection as previously described [47] and retrovirus packing was performed following the manufacturer's instructions (Thermo ScientificBio, Pittsburgh PA).

### Crystal Violet Assay

MCF-7-vector and MCF-7-miR-155 cells grown in 5% phenol free DMEM for 24 hours and then plated in 48 well plates (7000 cells per well) for 24 hours prior to a one time treatment with 1 nM E2 or DMSO. After 72 hours cells were washed once with PBS and fixed and stained using 0.1% Crystal Violet (in 20% methanol) for 10 minutes. Cells were washed with water and lysed with 1% SDS. Gene5 plate reader was used to read absorbance at wavelength 630. Each cell line was normalized to its respective DMSO treated group.

### Western blot analysis

MCF-7-vector and –miR-155 cells grown 10% FBS DMEM supplemented. Cells were washed with PBS and lysed with M-Per lysis buffer supplemented with 1% protease inhibitor and 1% phosphatase inhibitors (I/II) (Invitrogen, Grand Isles, NY). Supernatant containing protein extracts was obtained through centrifugation at 12,000 RPM for ten minutes at 4 degrees Celsius. Protein extracted per sample was determined by absorbance at 260 and 280 nm using the NanoDrop ND-1000. Proteins were heat denatured and 40ug of protein were loaded per lane on Bis-Tris-nuPAGE gel (Invitrogen, Grand Isles NY). Protein transfer to nitrocellulose through iBlot and iBlot transfer stacks as per manufacturer’s protocol (Invitrogen, Grand Isles, NY). Nonspecific binding was blocked by incubation in 5% BSA in 1% TBS-T for 1 hour. Overnight incubation of membrane with primary antibody for total mTOR, p-mTOR(S2448), Rictor, Raptor, TSC1, total p70s6kinase, p-4E-BP1(S65), AKT(S473), p-p70s6kinase (Thr389), p-S6 ribosomal protein (S235/236), p-eIF4B (S422), p-eEF2K (S366) diluted 1:1,000 (Cell Signaling Technology, Beverly MA) and PgR and ERα (Santa Cruz Biotechnology, Santa Cruz, CA) diluted 1:250 at 4 degree Celsius followed by three fifteen minute washes in 1% TBS-T. Membrane was incubated for 1 hour in secondary antibody 1:10,000 dilution (LiCor Bioscience, Lincoln NE) followed by three ten minute washes in 1% TBS-T. Band density was determined by LiCor gel imager. Normalization was to Rho GDIα Santa Cruz Biotechnology, Santa Cruz, CA) and images were cropped in Microsoft Photoshop.

### PRE-luciferase assay

Cells were plated in 24-well plates at a density of 5 × 10^5^ cells/well and allowed to attach overnight. After 18 hours, cells were transfected for 5 hours in serum free DMEM with 300 ng of PRE-luciferase plasmid, by using 6 to l of Effectene transfection reagent (QIAGEN, Valencia, CA) per microgram of DNA. After 5 hours, the transfection medium was removed and replaced with phenol red-free DMEM supplemented with 5% CSFBS containing vehicle, progesterone (100 nM, 50 nM, or 10 nM), or pretreated with 1 nM E2 for 30 minutes before treatment with progesterone (100 nM, 50 nM, or 10 nM) and incubated at 37°C. After 18 h, the medium was removed, and 100 ul of lysis buffer was added/well and incubated for 15 minutes at room temperature. Luciferase activity for the cell extracts was determined using luciferase substrate (Promega, Madison, WI) in an Auto Lumat Plus luminometer (Berthold, Oak Ridge, TN).

### Breast cancer data sources

Breast cancer gene expression deep sequencing was viewed through the University California Santa Cruz (UCSC) Cancer Genomics Browser and compiled by The Cancer Genome Atlas (TCGA) research network [18–21]. The TCGA dataset used was the breast invasive carcinoma and it was analyzed for gene expression aligned through the IlluminaHiSeq system with total tumor samples n = 1032 and gene signature used was receptor status (ERα).

Targeted gene expression correlation analysis for ER, PgR, Rictor, Rheb, and Raptor was derived from pooled breast cancer samples obtained from BC-GenExMiner-v3.0. Correlation maps were then generated based on breast cancer molecular subtype luminal A, Luminal B, and Basal-like. “Pooled” data refers to all data sets which were merged from all studies and converted to a common scale with normalization as per BC-GenExMiner-v3.0 designation [22, 48].

### Statistical analysis

Statistical Analysis was performed using Graph Pad Prism 5. Student’s *t* test was used to determine *p* values and statistically significant values had a *p* values of <0.05.

## Electronic supplementary material

Additional file 1: Figure S1: Pearson’s pairwise correlation for all breast cancer patients with a positive estrogen receptor status. Results obtained from Breast Cancer Gene-Expression Miner v3.0. (A) ERα and Rictor. N = 1,195 Pearson’s correlation coefficient (r) = 0.32 (B) ERα and Raptor. N = 1,220. Pearson’s correlation coefficient (r) = 0.20. (TIFF 340 KB)

Additional file 2: Table S1: Conserved miRNA predicted to target 8mer seed site in Rictor 3’UTR. (DOC 59 KB)

Additional file 3: Table S2: Conserved miRNA predicted to target 8mer seed site in Raptor 3’UTR. (DOC 31 KB)

Additional file 4: Figure S2: miR-155 Regulates Rictor Expression in breast cancer cell lines (A) QPCR for Rictor expression levels in MCF-7 cells stably transfected with miRNA predicted to target 3’UTR of Rictor. (B) qPCR for miR-155 expression in ER^-^ breast cancer cell lines, y-axis scaled to log scale. (C) qPCR for Rictor expression following stable transfection of miR-155 sponge or pmscv-vector in MDA-MB-157 cell line. (D) qPCR for miR-155 expression in MCF-7 cells stably transfected with pmscv-miR-155 or vector plasmid, y-axis scald to log scale. Error bars represent SEM. *** p < 0.001. (TIFF 820 KB)

Additional file 5: Figure S3: mTOR regulation of ER signaling in MCF-7-miR-155 cell line is not through direct phosphorylation of ERα MCF-7-miR-155 and MCF-7-vector cells were harvested for (A) qPCR for ERα expression levels and (B) western blot analysis of total ERα, phospho-ERα S167 and total PgR. Values normalized to Rho GDIα. Blot representative of three. (C) PRE-Luciferase was performed for MCF-7-vector and –miR-155 cells were treated with vehicle (DMSO) or progesterone in a dose dependent manner for 18 hours. (D) PRE-luciferase of MCF-7-vector and MCF-7-miR-155 cells pretreated with E2 (10 nM) for 30 minutes prior to 18 hours of stimulation with progesterone in a dose dependent manner. Bars represent fold change ± SEM of triplicate experiments. *, p < 0.05. (TIFF 923 KB)

Additional file 6: Figure S4: Rad001 inhibition of mTOR signaling in MCF-7-miR-155 cells. Western blot analysis of MCF-7-miR-155 for p-4E-BP1 and p-Akt S473 following 6hrs treatment with RAD001 (20 nM) or vehicle (DMSO). Blot representative of four. (TIFF 150 KB)

## Abbreviations

3’UTR: 3 Prime Untranslated Region
AKT: Cellular Homolog of Murine Thymoma Virus Akt8 Oncogene
AMPK: 5’AMP-Activated Protein Kinase
BCL2: B-Cell CLL/Lymphoma 2
DEPTOR: DEP Domain Containing mTOR-Interacting Protein
DMEM: Dulbecco’s Modified Eagle Medium
E2: 17β-Estradiol
ERα: Estrogen Receptor alpha
ERBB1: Epidermal Growth Factor Receptor
PRE: Progesterone Response Element
ERK1/2: Extracellular Signal Regulated Kinase 1/2
IGF: Insulin-Like Growth Factor
MAPK: Mitogen Activated Protein Kinase
MCF-7: Michigan Cancer Foundation 7
miRNA: Micro-Ribonucleic Acid
mTOR: Mammalian Target of Rapamycin
mTORC1: mTOR Complex 1
mTORC2: mTOR Complex 2
Ovex: Ovariectomized
PgR: Progesterone Receptor
PI3K: Phosphoinositol (PI) 3-Kinase
PLAU: Plasminogen Activator, Urokinase
qPCR: Quantitative Real Time RT-Polymerase Chain Reaction
RAD001: Everolimus
Raptor: Regulatory Associated Protein of TOR
Rictor: RPTOR Independent Companion of mTOR Complex 2
Rheb: Ras Homolog Enriched In Brain
p53: Tumor Protein p53
SDF-1: Stromal Cell-Derived Factor 1
SERPINA3: Serpin Peptidase Inhibitor, Clade A (Alpha-1 Antiproteinase
Antitrypsin: Member 3
TCGA: The Cancer Genome Atlas
TFF1: Trefoil Factor 1
TSC1: Tuberous Sclerosis Complex 1
TSC2: Tuberous Sclerosis Complex 2.

## References

[1] Dunlop EA, Tee AR (2009). Mammalian target of rapamycin complex 1: signalling inputs, substrates and feedback mechanisms. Cellular signalling.

[2] Dowling RJ, Topisirovic I, Alain T, Bidinosti M, Fonseca BD, Petroulakis E, Wang X, Larsson O, Selvaraj A, Liu Y, Kozma SC, Thomas G, Sonenberg N (2010). mTORC1-mediated cell proliferation, but not cell growth, controlled by the 4E-BPs. Science.

[3] Gulhati P, Bowen KA, Liu J, Stevens PD, Rychahou PG, Chen M, Lee EY, Weiss HL, O'Connor KL, Gao T, Evers BM (2011). mTORC1 and mTORC2 regulate EMT, motility, and metastasis of colorectal cancer via RhoA and Rac1 signaling pathways. Cancer research.

[4] Hay N, Sonenberg N (2004). Upstream and downstream of mTOR. Genes & development.

[5] Kim J, Kundu M, Viollet B, Guan KL (2011). AMPK and mTOR regulate autophagy through direct phosphorylation of Ulk1. Nature cell biology.

[6] Sengupta S, Peterson TR, Sabatini DM (2010). Regulation of the mTOR complex 1 pathway by nutrients, growth factors, and stress. Molecular cell.

[7] Huang J, Manning BD (2009). A complex interplay between Akt, TSC2 and the two mTOR complexes. Biochemical Society transactions.

[8] Huang J, Dibble CC, Matsuzaki M, Manning BD (2008). The TSC1-TSC2 complex is required for proper activation of mTOR complex 2. Molecular and cellular biology.

[9] Huang J, Wu S, Wu CL, Manning BD (2009). Signaling events downstream of mammalian target of rapamycin complex 2 are attenuated in cells and tumors deficient for the tuberous sclerosis complex tumor suppressors. Cancer research.

[10] Julien LA, Carriere A, Moreau J, Roux PP (2010). mTORC1-activated S6K1 phosphorylates Rictor on threonine 1135 and regulates mTORC2 signaling. Molecular and cellular biology.

[11] Carracedo A, Ma L, Teruya-Feldstein J, Rojo F, Salmena L, Alimonti A, Egia A, Sasaki AT, Thomas G, Kozma SC, Papa A, Nardella C, Cantley LC, Baselga J, Pandolfi PP (2008). Inhibition of mTORC1 leads to MAPK pathway activation through a PI3K-dependent feedback loop in human cancer. The Journal of clinical investigation.

[12] Feng Z, Zhang H, Levine AJ, Jin S (2005). The coordinate regulation of the p53 and mTOR pathways in cells. Proceedings of the National Academy of Sciences of the United States of America.

[13] Cui X, Schiff R, Arpino G, Osborne CK, Lee AV (2005). Biology of progesterone receptor loss in breast cancer and its implications for endocrine therapy. Journal of clinical oncology : official journal of the American Society of Clinical Oncology.

[14] Cui X, Zhang P, Deng W, Oesterreich S, Lu Y, Mills GB, Lee AV (2003). Insulin-like growth factor-I inhibits progesterone receptor expression in breast cancer cells via the phosphatidylinositol 3-kinase/Akt/mammalian target of rapamycin pathway: progesterone receptor as a potential indicator of growth factor activity in breast cancer. Molecular endocrinology.

[15] Becker MA, Ibrahim YH, Cui X, Lee AV, Yee D (2011). The IGF pathway regulates ERalpha through a S6K1-dependent mechanism in breast cancer cells. Molecular endocrinology.

[16] Boulay A, Rudloff J, Ye J, Zumstein-Mecker S, O'Reilly T, Evans DB, Chen S, Lane HA (2005). Dual inhibition of mTOR and estrogen receptor signaling in vitro induces cell death in models of breast cancer. Clinical cancer research : an official journal of the American Association for Cancer Research.

[17] Chang SB, Miron P, Miron A, Iglehart JD (2007). Rapamycin inhibits proliferation of estrogen-receptor-positive breast cancer cells. The Journal of surgical research.

[18] Goldman M, Craft B, Swatloski T, Ellrott K, Cline M, Diekhans M, Ma S, Wilks C, Stuart J, Haussler D, Zhu J (2013). The UCSC Cancer Genomics Browser: update 2013. Nucleic acids research.

[19] Sanborn JZ, Benz SC, Craft B, Szeto C, Kober KM, Meyer L, Vaske CJ, Goldman M, Smith KE, Kuhn RM, Karolchik D, Kent WJ, Stuart JM, Haussler D, Zhu J (2011). The UCSC Cancer Genomics Browser: update 2011. Nucleic acids research.

[20] Vaske CJ, Benz SC, Sanborn JZ, Earl D, Szeto C, Zhu J, Haussler D, Stuart JM (2010). Inference of patient-specific pathway activities from multi-dimensional cancer genomics data using PARADIGM. Bioinformatics.

[21] Zhu J, Sanborn JZ, Benz S, Szeto C, Hsu F, Kuhn RM, Karolchik D, Archie J, Lenburg ME, Esserman LJ, Kent WJ, Haussler D, Wang T (2009). The UCSC Cancer Genomics Browser. Nature methods.

[22] Jezequel P, Frenel JS, Campion L, Guerin-Charbonnel C, Gouraud W, Ricolleau G, Campone M (2013). bc-GenExMiner 3.0: new mining module computes breast cancer gene expression correlation analyses. Database: the journal of biological databases and curation.

[23] Martin EC, Bratton MR, Zhu Y, Rhodes LV, Tilghman SL, Collins-Burow BM, Burow ME (2012). Insulin-like growth factor-1 signaling regulates miRNA expression in MCF-7 breast cancer cell line. PloS one.

[24] Schaefer CF, Anthony K, Krupa S, Buchoff J, Day M, Hannay T, Buetow KH (2009). PID: the Pathway Interaction Database. Nucleic acids research.

[25] Chen X, Zhao M, Hao M, Sun X, Wang J, Mao Y, Zu L, Liu J, Shen Y, Wang J, Shen K (2013). Dual inhibition of PI3K and mTOR mitigates compensatory AKT activation and improves tamoxifen response in breast cancer. Molecular cancer research : MCR.

[26] Rhead B, Karolchik D, Kuhn RM, Hinrichs AS, Zweig AS, Fujita PA, Diekhans M, Smith KE, Rosenbloom KR, Raney BJ, Pohl A, Pheasant M, Meyer LR, Learned K, Hsu F, Hillman-Jackson J, Harte RA, Giardine B, Dreszer TR, Clawson H, Barber GP, Haussler D, Kent WJ (2010). The UCSC Genome Browser database: update 2010. Nucleic acids research.

[27] Wang ET, Sandberg R, Luo S, Khrebtukova I, Zhang L, Mayr C, Kingsmore SF, Schroth GP, Burge CB (2008). Alternative isoform regulation in human tissue transcriptomes. Nature.

[28] Creighton CJ (2012). The molecular profile of luminal B breast cancer. Biologics: targets & therapy.

[29] Kim HJ, Cui X, Hilsenbeck SG, Lee AV (2006). Progesterone receptor loss correlates with human epidermal growth factor receptor 2 overexpression in estrogen receptor-positive breast cancer. Clinical cancer research: an official journal of the American Association for Cancer Research.

[30] Lu Z, Ye Y, Jiao D, Qiao J, Cui S (2012). Liu Z: miR-155 and miR-31 are differentially expressed in breast cancer patients and are correlated with the estrogen receptor and progesterone receptor status. Oncology letters.

[31] Chen CC, Jeon SM, Bhaskar PT, Nogueira V, Sundararajan D, Tonic I, Park Y, Hay N (2010). FoxOs inhibit mTORC1 and activate Akt by inducing the expression of Sestrin3 and Rictor. Developmental cell.

[32] Sarbassov DD, Ali SM, Kim DH, Guertin DA, Latek RR, Erdjument-Bromage H, Tempst P, Sabatini DM (2004). Rictor, a novel binding partner of mTOR, defines a rapamycin-insensitive and raptor-independent pathway that regulates the cytoskeleton. Current biology : CB.

[33] Jordan VC, Lewis-Wambi JS, Patel RR, Kim H, Ariazi EA (2009). New hypotheses and opportunities in endocrine therapy: amplification of oestrogen-induced apoptosis. Breast.

[34] Liu WH, Yeh SH, Lu CC, Yu SL, Chen HY, Lin CY, Chen DS, Chen PJ (2009). MicroRNA-18a prevents estrogen receptor-alpha expression, promoting proliferation of hepatocellular carcinoma cells. Gastroenterology.

[35] Rao X, Di Leva G, Li M, Fang F, Devlin C, Hartman-Frey C, Burow ME, Ivan M, Croce CM, Nephew KP (2011). MicroRNA-221/222 confers breast cancer fulvestrant resistance by regulating multiple signaling pathways. Oncogene.

[36] Zhao JJ, Lin J, Yang H, Kong W, He L, Ma X, Coppola D, Cheng JQ (2008). MicroRNA-221/222 negatively regulates estrogen receptor alpha and is associated with tamoxifen resistance in breast cancer. The Journal of biological chemistry.

[37] Zhang C, Zhao J, Deng H (2013). 17beta-estradiol up-regulates miR-155 expression and reduces TP53INP1 expression in MCF-7 breast cancer cells. Molecular and cellular biochemistry.

[38] Jiang S, Zhang HW, Lu MH, He XH, Li Y, Gu H, Liu MF, Wang ED (2010). MicroRNA-155 functions as an OncomiR in breast cancer by targeting the suppressor of cytokine signaling 1 gene. Cancer research.

[39] Kong W, He L, Coppola M, Guo J, Esposito NN, Coppola D, Cheng JQ (2010). MicroRNA-155 regulates cell survival, growth, and chemosensitivity by targeting FOXO3a in breast cancer. The Journal of biological chemistry.

[40] Wang F, Zheng Z, Guo J, Ding X (2010). Correlation and quantitation of microRNA aberrant expression in tissues and sera from patients with breast tumor. Gynecologic oncology.

[41] Wang J, Yang K, Zhou L, Minhaowu A, Wu Y, Zhu M, Lai X, Chen T, Feng L, Li M, Huang C, Zhong Q, Huang X (2013). MicroRNA-155 promotes autophagy to eliminate intracellular mycobacteria by targeting Rheb. PLoS pathogens.

[42] Wan G, Xie W, Liu Z, Xu W, Lao Y, Huang N, Cui K, Liao M, He J, Jiang Y, Yang BB, Xu H, Xu N, Zhang Y (2014). Hypoxia-induced MIR155 is a potent autophagy inducer by targeting multiple players in the MTOR pathway. Autophagy.

[43] Salvo VA, Boue SM, Fonseca JP, Elliott S, Corbitt C, Collins-Burow BM, Curiel TJ, Srivastav SK, Shih BY, Carter-Wientjes C, Wood CE, Erhardy PW, Beckman BS, McLachlan JA, Cleveland TE, Burow ME (2006). Antiestrogenic glyceollins suppress human breast and ovarian carcinoma tumorigenesis. Clinical cancer research : an official journal of the American Association for Cancer Research.

[44] Schmittgen TD, Zakrajsek BA, Mills AG, Gorn V, Singer MJ, Reed MW (2000). Quantitative reverse transcription-polymerase chain reaction to study mRNA decay: comparison of endpoint and real-time methods. Analytical biochemistry.

[45] Yin Q, McBride J, Fewell C, Lacey M, Wang X, Lin Z, Cameron J, Flemington EK (2008). MicroRNA-155 is an Epstein-Barr virus-induced gene that modulates Epstein-Barr virus-regulated gene expression pathways. Journal of virology.

[46] Marsolier J, Pineau S, Medjkane S, Perichon M, Yin Q, Flemington E, Weitzman MD, Weitzman JB (2013). OncomiR addiction is generated by a miR-155 feedback loop in Theileria-transformed leukocytes. PLoS pathogens.

[47] Tate CR, Rhodes LV, Segar HC, Driver JL, Pounder FN, Burow ME, Collins-Burow BM (2012). Targeting triple-negative breast cancer cells with the histone deacetylase inhibitor panobinostat. Breast cancer research: BCR.

[48] Jezequel P, Campone M, Gouraud W, Guerin-Charbonnel C, Leux C, Ricolleau G (2012). Campion L: bc-GenExMiner: an easy-to-use online platform for gene prognostic analyses in breast cancer. Breast cancer research and treatment.

