# Supplementary figures and images for: microRNA regulation of mammalian target of rapamycin expression and activity controls estrogen receptor function and RAD001 sensitivity
Source: Mol Cancer. 2014 Oct 6;13:229. doi: 10.1186/1476-4598-13-229 (PMC4203920; doi:10.1186/1476-4598-13-229)

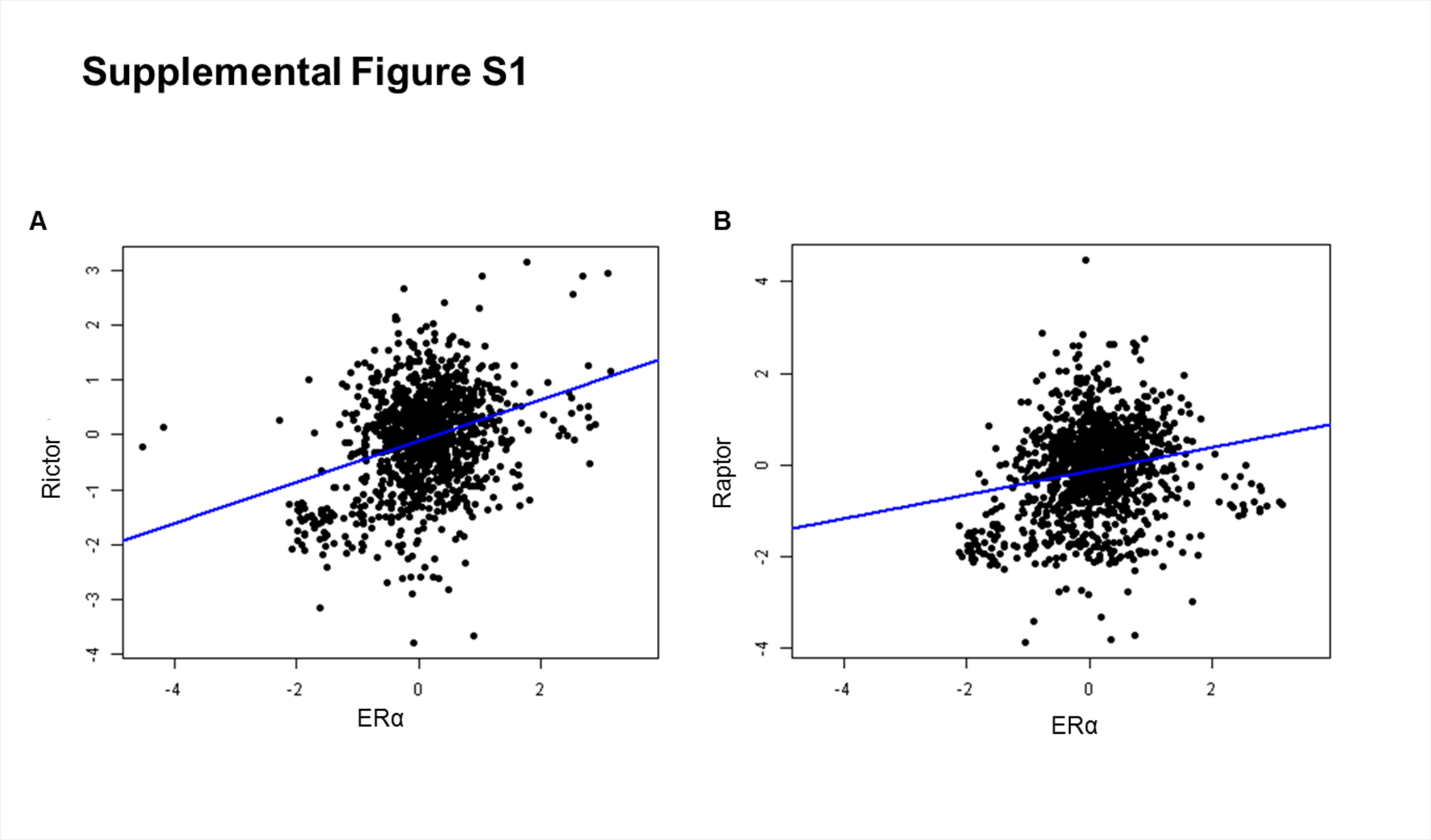

Supplement: Supplementary file 1 — Additional file 1: Figure S1: Pearson’s pairwise correlation for all breast cancer patients with a positive estrogen receptor status. Results obtained from Breast Cancer Gene-Expression Miner v3.0. (A) ERα and Rictor. N = 1,195 Pearson’s correlation coefficient (r) = 0.32 (B) ERα and Raptor. N = 1,220. Pearson’s correlation coefficient (r) = 0.20. (TIFF 340 KB) [file 12943_2014_1435_MOESM1_ESM.tiff]

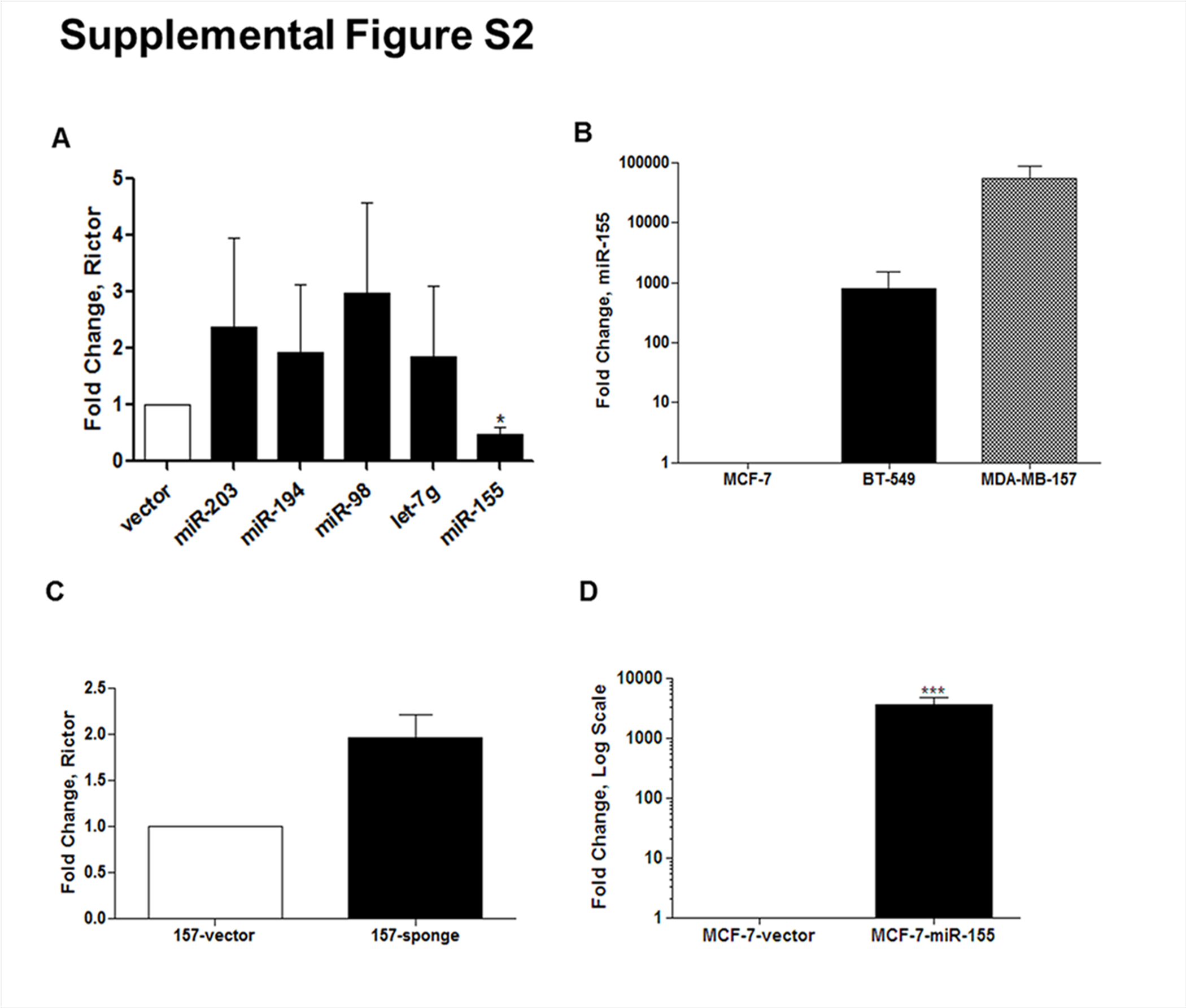

Supplement: Supplementary file 4 — Additional file 4: Figure S2: miR-155 Regulates Rictor Expression in breast cancer cell lines (A) QPCR for Rictor expression levels in MCF-7 cells stably transfected with miRNA predicted to target 3’UTR of Rictor. (B) qPCR for miR-155 expression in ER- breast cancer cell lines, y-axis scaled to log scale. (C) qPCR for Rictor expression following stable transfection of miR-155 sponge or pmscv-vector in MDA-MB-157 cell line. (D) qPCR for miR-155 expression in MCF-7 cells stably transfected with pmscv-miR-155 or vector plasmid, y-axis scald to log scale. Error bars represent SEM. *** p < 0.001. (TIFF 820 KB) [file 12943_2014_1435_MOESM4_ESM.tiff]

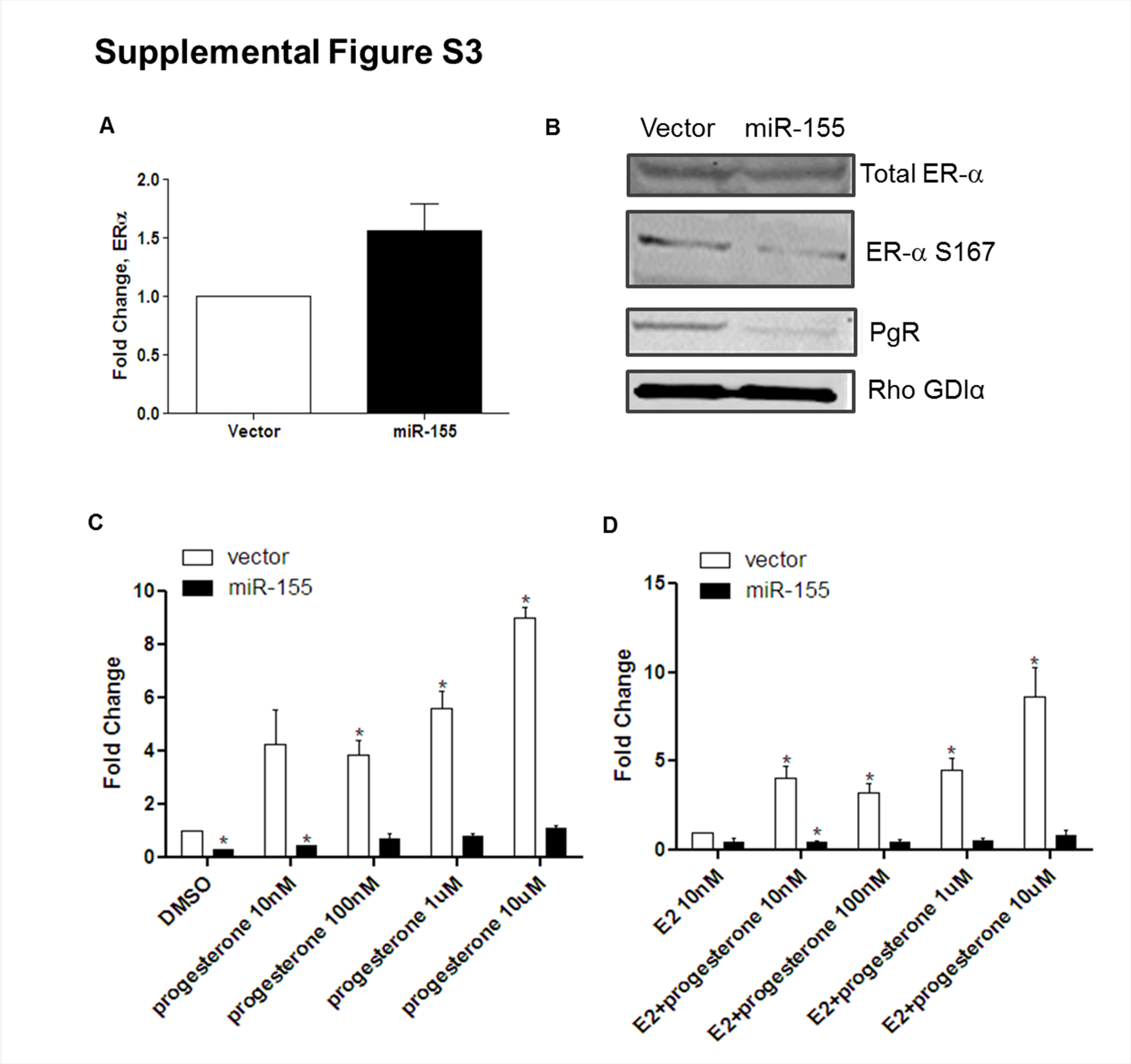

Supplement: Supplementary file 5 — Additional file 5: Figure S3: mTOR regulation of ER signaling in MCF-7-miR-155 cell line is not through direct phosphorylation of ERα MCF-7-miR-155 and MCF-7-vector cells were harvested for (A) qPCR for ERα expression levels and (B) western blot analysis of total ERα, phospho-ERα S167 and total PgR. Values normalized to Rho GDIα. Blot representative of three. (C) PRE-Luciferase was performed for MCF-7-vector and –miR-155 cells were treated with vehicle (DMSO) or progesterone in a dose dependent manner for 18 hours. (D) PRE-luciferase of MCF-7-vector and MCF-7-miR-155 cells pretreated with E2 (10 nM) for 30 minutes prior to 18 hours of stimulation with progesterone in a dose dependent manner. Bars represent fold change ± SEM of triplicate experiments. *, p < 0.05. (TIFF 923 KB) [file 12943_2014_1435_MOESM5_ESM.tiff]

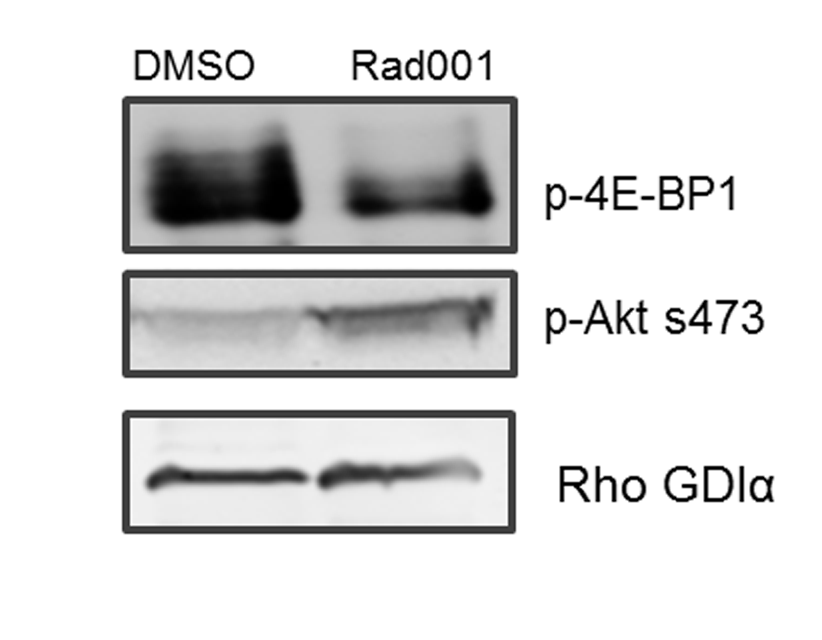

Supplement: Supplementary file 6 — Additional file 6: Figure S4: Rad001 inhibition of mTOR signaling in MCF-7-miR-155 cells. Western blot analysis of MCF-7-miR-155 for p-4E-BP1 and p-Akt S473 following 6hrs treatment with RAD001 (20 nM) or vehicle (DMSO). Blot representative of four. (TIFF 150 KB) [file 12943_2014_1435_MOESM6_ESM.tiff]
