# Supplementary material for: microRNA regulation of mammalian target of rapamycin expression and activity controls estrogen receptor function and RAD001 sensitivity
Source: Mol Cancer. 2014 Oct 6;13:229. doi: 10.1186/1476-4598-13-229 (PMC4203920; doi:10.1186/1476-4598-13-229)
Supplement: Supplementary file 2 — Additional file 2: Table S1: Conserved miRNA predicted to target 8mer seed site in Rictor 3’UTR. (DOC 59 KB) [file 12943_2014_1435_MOESM2_ESM.doc]

**Additional file 2: Table S1 Conserved miRNA predicted to target 8mer seed site in Rictor 3’UTR**

| miRNA | Total | 8mer | 7mer-m8 | 7mer-1A |
| --- | --- | --- | --- | --- |
| miR-142-3p | 3 | 0 | 2 | 1 |
| miR-19ab | 2 | 1 | 0 | 1 |
| miR-96/507/1271 | 2 | 0 | 1 | 1 |
| miR-155 | 2 | 1 | 0 | 1 |
| miR-148ab-3p/152 | 2 | 0 | 1 | 1 |
| miR-15abc/16/16abc/195/322/ 424/497/1907 | 2 | 0 | 1 | 1 |
| miR-218/218a | 2 | 0 | 1 | 1 |
| miR-153 | 2 | 0 | 0 | 2 |
| miR-33a-3p/365/365-3p | 1 | 1 | 0 | 0 |
| miR-503 | 1 | 1 | 0 | 0 |
| miR-204/204b/211 | 1 | 0 | 1 | 0 |
| miR-192/215 | 1 | 0 | 1 | 0 |
| let-7/98/4458/4500 | 1 | 1 | 0 | 0 |
| miR-182 | 1 | 0 | 0 | 1 |
| miR-135ab/135a-5p | 1 | 0 | 0 | 1 |
| miR-1ab/206/613 | 1 | 1 | 0 | 0 |
| miR-137/137ab | 1 | 1 | 0 | 0 |
| miR-217 | 1 | 0 | 1 | 0 |
| miR-143/1721/4770 | 1 | 1 | 0 | 0 |
| miR-196abc | 1 | 0 | 1 | 0 |
| miR-128/128ab | 1 | 0 | 1 | 0 |
| miR-129-5p/129ab-5p | 1 | 0 | 0 | 1 |
| miR-133abc | 1 | 0 | 0 | 1 |
| miR-194 | 3 | 0 | 2 | 1 |
| miR-203 | 3 | 0 | 2 | 1 |
