# Supplementary material for: microRNA regulation of mammalian target of rapamycin expression and activity controls estrogen receptor function and RAD001 sensitivity
Source: Mol Cancer. 2014 Oct 6;13:229. doi: 10.1186/1476-4598-13-229 (PMC4203920; doi:10.1186/1476-4598-13-229)
Supplement: Supplementary file 3 — Additional file 3: Table S2: Conserved miRNA predicted to target 8mer seed site in Raptor 3’UTR. (DOC 31 KB) [file 12943_2014_1435_MOESM3_ESM.doc]

**Additional file 3: Table S2** Conserved miRNA predicted to target 8mer seed site in Raptor 3’UTR

| miRNA | Total | 8mer | 7mer-m8 | 7mer-1A |
| --- | --- | --- | --- | --- |
| miR-23abc/23b-3p | 1 | 1 | 0 | 0 |
| miR-155 | 1 | 0 | 1 | 0 |
